# Chlorella intake attenuates reduced salivary SIgA secretion in *kendo* training camp participants

**DOI:** 10.1186/1475-2891-11-103

**Published:** 2012-12-11

**Authors:** Takeshi Otsuki, Kazuhiro Shimizu, Motoyuki Iemitsu, Ichiro Kono

**Affiliations:** 1 Faculty of Sport and Health Sciences, Ryutsu Keizai University, Ryugasaki, Ibaraki, Japan; 2 Sports Research & Development Core, University of Tsukuba, Tsukuba, Ibaraki, Japan; 3 Faculty of sport and health science, Ritsumeikan University, Kusatsu, Shiga, Japan; 4 University of Tsukuba, Tsukuba, Ibaraki, Japan

**Keywords:** IgA, Immunological depression, *Kendo*, Multicomponent supplement

## Abstract

**Background:**

The green alga Chlorella contains high levels of proteins, vitamins, and minerals. We previously reported that a chlorella-derived multicomponent supplement increased the secretion rate of salivary secretory immunoglobulin A (SIgA) in humans. Here, we investigated whether intake of this chlorella-derived supplement attenuated the reduced salivary SIgA secretion rate during a *kendo* training camp.

**Methods:**

Ten female *kendo* athletes participated in inter-university 6-day spring and 4-day summer camps. They were randomized into two groups; one took placebo tablets during the spring camp and chlorella tablets during the summer camp, while the other took chlorella tablets during the spring camp and placebo tablets during the summer camp. Subjects took these tablets starting 4 weeks before the camp until post-camp saliva sampling. Salivary SIgA concentrations were measured by ELISA.

**Results:**

All subjects participated in nearly all training programs, and body-mass changes and subjective physical well-being scores during the camps were comparable between the groups. However, salivary SIgA secretion rate changes were different between these groups. Salivary SIgA secretion rates decreased during the camp in the placebo group (before vs. second, middle, and final day of camp, and after the camp: 146 ± 89 vs. 87 ± 56, 70 ± 45, 94 ± 58, and 116 ± 71 μg/min), whereas no such decreases were observed in the chlorella group (121 ± 53 vs. 113 ± 68, 98 ± 69,115 ± 80, and 128 ± 59 μg/min).

**Conclusion:**

Our results suggest that a use of a chlorella-derived dietary supplement attenuates reduced salivary SIgA secretion during a training camp for a competitive sport.

## Introduction

Salivary secretory immunoglobulin A (SIgA) plays a crucial role in mucosal immune function and is the first line of defense against pathogenic microbial invasion in humans [1]. Rounds of heavy exercise depress salivary SIgA secretion, which results in an increased risk of infection [2,3]. In particular, athletes appear to require infection control during training camps because of the high-intensity physical activity and group living. Yamauchi *et al.*[4] demonstrated that the secretion rate of salivary SIgA decreased by approximately 25% during a rugby football training camp, and this decrease was inversely related to the number of upper respiratory symptoms. Akimoto *et al.*[5] also reported that the salivary SIgA secretion rate decreased by approximately 45% during a 3-day soccer competition involving 6 games. These reductions in salivary SIgA secretion in athletes may be preventable with dietary supplements. Indeed, various dietary supplements (*e.g.*, vitamins, bovine colostrum, and probiotics) have been tested for their effects on attenuating or suppressing the intensive exercise-related declines in immune function. However, the recently published position statement of the International Society of Exercise and Immunology noted that no adequate dietary supplements for SIgA secretion have been proposed for athletes [6].

Chlorella is a unicellular green alga that grows in fresh water and contains high levels of proteins, chlorophylls, vitamins, minerals, and dietary fibers. Previous studies showed that chlorella-derived dietary supplements improved SIgA concentrations in breast milk [7] and antibody responses to influenza in subjects aged 50–55 years [8]. Based on these findings, we conducted an intervention study and demonstrated that a 4-week chlorella supplementation program increased the salivary SIgA secretion rate by 40% in healthy humans [9]. However, it is not known if chlorella intake can attenuate reduced salivary SIgA secretion during a training camp for a competitive sport.

We hypothesized that a chlorella-derived dietary supplement would attenuate reduced salivary SIgA secretion during a training camp for a competitive sport. To test this hypothesis, we investigated the effects of a placebo and a chlorella supplement on the salivary SIgA secretion of participants during a training camp for *kendo*, a traditional Japanese sport, by using a single blinded, placebo-control study design. Although the absolute concentration of salivary SIgA is a good marker of mucosal immune function, a dehydration-induced reduction in the saliva flow rate can result in a sham increase in the SIgA concentration during a training camp. Therefore, we used the salivary SIgA secretion rate (*i.e.*, the total secretion volume per unit time) as the marker, as in previous studies [2,4,5,9]. Additionally, we compared salivary SIgA concentrations and saliva flow rates as indicators of possible mechanisms underlying the changes in the salivary SIgA secretion rate (*i.e.*, SIgA secretory function of T-cells and SIgA transport function by saliva). *Kendo* is a martial art in which a bamboo sword and protective armor *bogu*; *men* (mask), *do* (breastplate), *kote* (gloves), and *tare* (groin protector)] are used. The basic techniques are striking (*men*, *kote,* and *do*) and frontal thrusts to the neck. A match comprises a duel between two athletes and is the best of three points lasting up to 5 min. Because the match and practice include face-to-face contact, a *kendo* tournament is a risk factor for infections [10]. There is also evidence that a *kendo* training camp involves high-intensity exercise, which reduces the levels of immune function-related cells including lymphocytes and other white blood cells [11,12]. Therefore, we recruited *kendo* athletes as subjects for this study.

## Materials and methods

### Participants

Ten female *kendo* athletes who belonged to a national-level university team participated in this study. Their usual training time and frequency were 120 min/day and 6 days/week. The mean rating of perceived exertion (Borg’s 6-20 scale) was 13.6 ± 1.2. None of these athletes used dietary supplements on a regular basis and had no signs, symptoms, or history of overt chronic diseases. The participants were asked not to change their regular lifestyles (*e.g.*, eating habits) while taking the placebo or chlorella tablets during the experimental period. Their mean age was 20.1 ± 0.9 years and their mean height was 1.58 ± 0.04 m.

This study was approved by the Ethical Committee of the Institute of Health and Sport Sciences of the University of Tsukuba and conformed to the principles of the Helsinki Declaration. All participants gave their written informed consent prior to study initiation.

### Experimental design

The subjects participated in inter-university *kendo* training camps during March (6-day spring camp) and August (4-day summer camp). The subjects were randomized into two groups; one group took placebo tablets during the spring camp and chlorella tablets (SunChlorella A; SunChlorella, Kyoto, Japan) during the summer camp, and the other group took chlorella tablets during the spring camp and placebo tablets during the summer camp. Four weeks before the spring camp, each group was prescribed either placebo or chlorella tablets in a single-blinded manner, which were to be taken at 30 tablets per day (15 tablets × twice, after breakfast and dinner). This was in accord with a general prescription for Japanese consumers of this product.

Based on the methods used in previous studies [5,9,13,14], saliva samples were obtained four days before the camp, on the second, fourth (middle), and sixth (final) day of the camp, and four days after the spring camp. Tablet intake was stopped after the post-camp saliva sample was collected. Patient compliance with regard to regular intake was documented in intake logs. Fifteen weeks after the end of the spring camp (*i.e.*, after a 15-week washout period), the summer camp trial was commenced. The procedures were the same as those used for the spring camp trial with a few modifications; saliva samples were collected one day before the camp; on the second, third (middle), and fourth (final) day of the camp; and five days after the camp.

Saliva samples before the spring camp were collected in the morning after overnight fasting. Sampling before the summer camp was done before lunch; subjects were asked to refrain from drinking and eating without water between the end of breakfast and saliva sampling. The time from the last training session to these saliva samplings was >20 h. During the spring and summer camps, saliva samples were collected as soon as possible after the end of the morning training session (*i.e.*, within 60 min). Again, subjects were asked to refrain from drinking and eating without water from the end of breakfast to the sampling. The after-camp saliva samplings were done in the morning after overnight fasting; the time from the last training session was >20 h.

### Training camp

The *kendo* training camps included two separate training sessions on each day, one in the morning for 3.0 h (0900–1200) during both camps and one in the afternoon for 4.0 h during the spring camp (1300–1700) and 3.0 h during the summer camp (1400–1700). There were no morning sessions on the first day of both camps. These sessions comprised warming-up periods, *kendo* practices, games, breaks, and cooling-down periods. Practice sessions consisted of *kihon-keiko* (practice to acquire basic movements), *gokaku-keiko* (*keiko* practiced by persons of nearly equal skill), and *kakari-keiko* (*keiko* method: for a short period, the trainee practices striking the *motodachi*, person acting as an instructor, with all learned *waza* techniques without concern for being struck or dodging strikes). Previous studies reported that the percentages of maximal oxygen uptake for *kihon-keiko*, *gokaku-keiko*, *kakari-keiko*, and an entire session including warming-up and breaks were approximately 40%, 55%, 70%, and 45–55%, respectively [15,16].

### Placebo and chlorella tablets

The placebo and chlorella tablets used in this study were the same as those used in our previous study [9]. The mass of each placebo and chlorella tablet was 243 mg and 200 mg, respectively. The main components of the placebo tablet were lactose and a colorant. The chlorella tablet comprised dried chlorella pyrenoidosa powder as the main ingredient. The respective nutritional values per 100 g of the placebo and chlorella tables were: energy, 406 and 399 kcal; moisture, 3.2 and 5.3 g; protein, 2.0 and 60.8 g; lipid, 5.9 and 9.2 g; saccharide, 85.6 and 6.3 g; dietary fiber, 1.1 and 11.9 g; and ash, 2.2 and 6.5 g. Additional multicomponent analysis of the chlorella-derived tablets was made by the Japan Dairy Technical Association (Tokyo, Japan).

### Saliva samples

Saliva samples were obtained and analyzed in duplicate as described previously [5,9,13,14]. The inter-assay coefficient of variation for this method was reported to be 6.2% [13]. Briefly, the subjects rinsed their mouths with distilled water (three times for 30 s) and then rested for 5 min. Saliva production was stimulated by chewing sterilized cotton (Salivette; Sarstedt, Nümbrecht, Germany) at a frequency of 60 chews/60 s. Based on previous studies, the amount of saliva in grams was converted to milliliters assuming a saliva density of 1 g/mL. The saliva sample obtained was separated from the cotton by centrifugation at 1,460 *g*. After measuring the sample volumes, samples were frozen at −60°C. We measured salivary SIgA concentrations using an enzyme-linked immunosorbent assay, as in previous studies [5,9,13,14]. The SIgA secretion rate (*μ*g/min) was obtained from the product of the absolute SIgA concentration (*μ*g/mL) and the saliva flow rate (mL/min).

### Subjective rating of physical well-being

Before saliva sampling, the subjects recorded the following subjective ratings: lightness of body, fatigue, and muscle tension. These ratings were subjectively assessed on a scale of 1 (light), 2, 3 (usual), 4, and 5 (heavy) by the subjects.

### Statistical analysis

Results are given as means ± SDs. Inter-group comparisons for patient compliance and measured variables (body weight, SIgA secretion rate and concentration, saliva flow rate, and subjective ratings of physical well-being) before the camp were made by unpaired *t-*tests. Two-way, repeated measures analysis of variance (ANOVA) was used to assess the effects of the camps. If a significant *F* value was found, a post hoc test using Fisher’s protected least significant differences was done. When a variable was not normally distributed based on a Kolmogorov-Smirnov test, the comparison with before camp was made using a non-parametric Wilcoxon's rank test. *P*-values of < 0.05 were considered significant. All statistical analyses used StatView statistical software (SAS Institute, Inc., Cary, NC).

## Results

Most of the recorded variables were normally distributed, although the subjective ratings of physical well-being on the second, middle, and final days of the camps were non-normally distributed in both groups. There were no differences in these indices before the camps between the placebo and chlorella groups.

**Figure 1:**
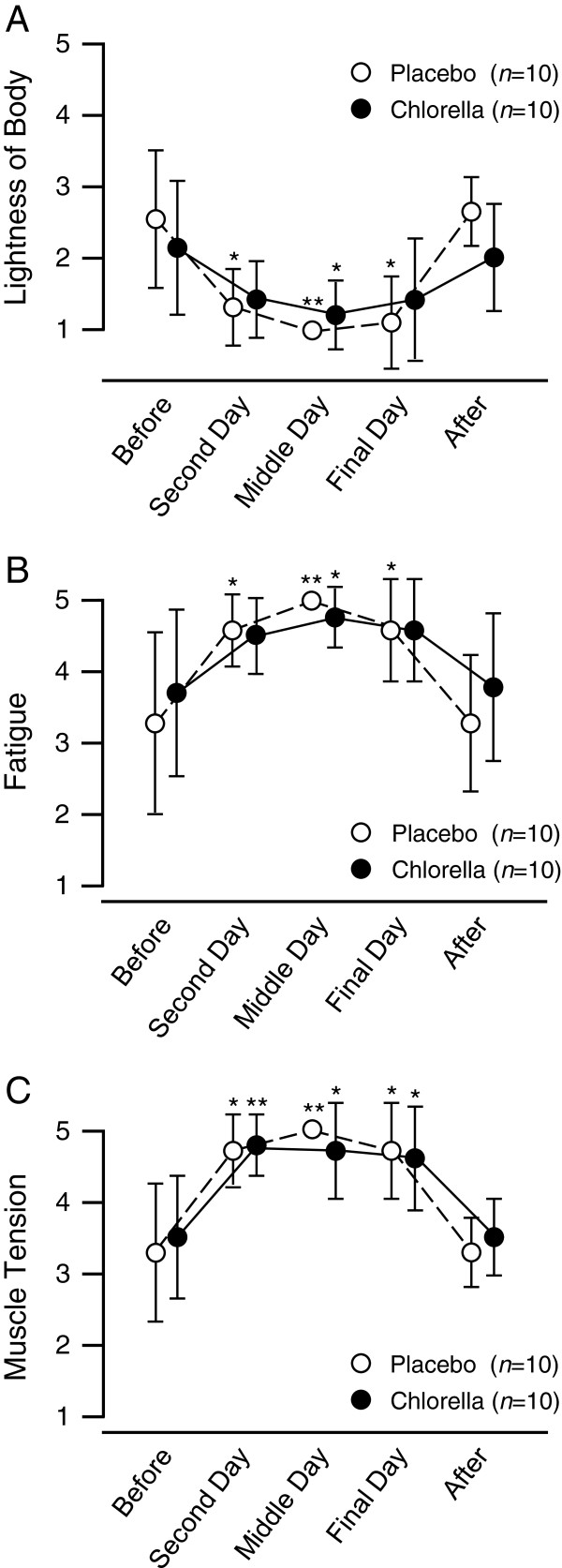
**Subjective ratings of physical well-being during the *kend*o training camps.** Values are means ± SDs. *, *P* < 0.05 vs. before the training camp; **, *P* < 0.01 vs. before the training camp.

Compliance with respect to regular tablet intake was comparable between the placebo (96.8 ± 3.2%) and the chlorella (94.1 ± 6.9%) groups. There was no effect of the *kendo* training camp on body mass (Table 1). The subjective ratings of physical well-being are shown in Figure 1. The scores for lightness of body decreased (Figure 1A) and those for fatigue (Figure 1B) and muscle tension (Figure 1C) increased during the camps compared with baseline in both groups. The changes in lightness of body at the second day and fatigue at the second and final days in the chlorella group were not statistically significant, although the *P*-values were close to a significant level (0.06, 0.07, and 0.07, respectively). No subjects reported any subjective upper respiratory symptoms during the training camps. 

**Table 1:** **Body mass during training camps in *kendo *athletes**

		**Placebo (*n*** **= 10)**			**Chlorella (*n*** **= 10)**		
Before		57.9	±	5.1 kg	57.8	±	5.7 kg
Camp	Second Day	58.3	±	5.5 kg	57.8	±	5.4 kg
	Middle Day	58.3	±	5.4 kg	58.0	±	5.7 kg
	Final Day	58.5	±	5.5 kg	58.2	±	5.9 kg
After		57.9	±	5.3 kg	58.0	±	5.3 kg

Values are means ± SDs.

**Figure 2:**
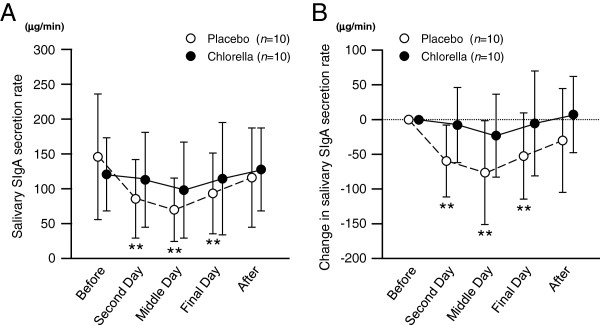
**Salivary secretory immunoglobulin A (SIgA) secretion rates during the k*end*o training camps.** Values are means ± SDs. **, *P* < 0.01 vs. before the training camp.

**Figure 3:**
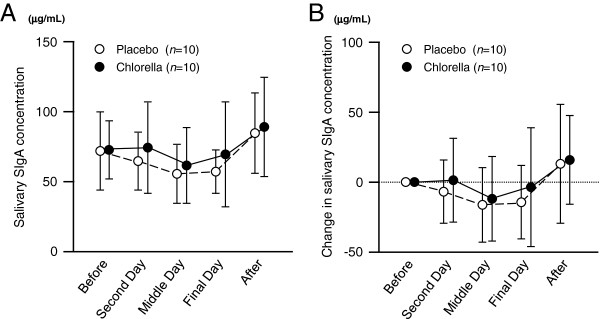
**Salivary secretory immunoglobulin A (SIgA) concentrations during the k*endo *training camps.** Values are means ± SDs.

**Figure 4:**
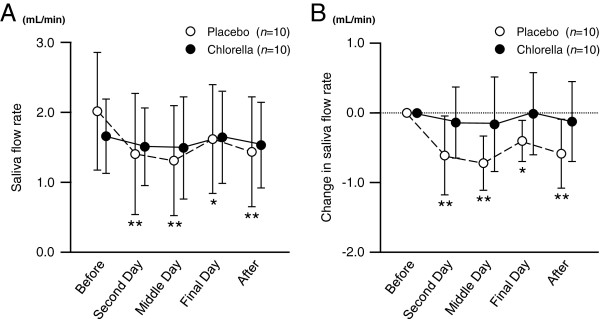
**Saliva flow rates during the k*endo *training camps.** Values are means ± SDs. *, *P* < 0.05 vs. before training camp; **, *P* < 0.01 vs. before training camp.

Figure 2 shows the salivary SIgA secretion rates during the training camps. The salivary SIgA secretion rates during the camp with placebo intake were lower than baseline, but there were no differences between before and during the camp in the chlorella group. There were no differences in the salivary SIgA concentrations between before and during the camp in both the placebo and chlorella groups (Figure 3A). Also with respect to changes in salivary SIgA concentrations, no differences were detected between before and during the camp (Figure 3B). The saliva flow rate decreased during and after the camp in the placebo group compared to the pre-camp level, whereas it did not change in the chlorella group (Figure 4). 

Numerous nutrients were detected by the multicomponent analysis of the chlorella-derived tablets. The nutrients that showed possible immune-enhancing effects in previous studies are shown in Table 2. 

**Table 2:** Nutrients detected by multicomponent analysis of chlorella-derived tablet

**Total**		**Detail**	
** Nutrient**	**Value**	**Nutrient**	**Value**
Protein, g/100g	60.8	Valine, g/100g	3.10
		Leucine, g/100g	4.37
		Isoleucine, g/100g	2.04
Lipid, g/100g	9.2	n-3 unsaturated fatty acid, %	8.6
Ash, g/100g	6.5	Vitamin A (retinol equivalent), *μ*g/100g	454
		Vitamin B6, mg/100g	2.1
		Vitamin C, mg/100g	4.0
		Pantothenic acid, mg/100g	4.9
		Folate, mg/100g	2.4
		Iron, mg/100g	140

Numerous nutrients were detected by multicomponent analysis of chlorella-derived tablets. The nutrients that showed possible immune-enhancing effects in previous studies are shown in this table [6,17-24].

## Discussion

We investigated the effects of placebo and chlorella intake on salivary SIgA secretion in the participants of a *kendo* training camp. Although the scores for physical well-being before the camp were slightly lower than a usual level (3.0), all of our subjects participated in nearly all of the training programs. Body mass changes and subjective physical well-being scores during the camp were comparable between the placebo and chlorella groups. Based on these results, we consider that there were no intergroup differences in the physical requirements of this training camp. However, with respect to the baseline levels, salivary SIgA secretion rates decreased only with placebo intake but not with chlorella supplementation. These changes in the salivary SIgA secretion rate were comparable to the changes in the saliva flow rate. There were no changes in salivary SIgA concentrations during the camp. These results suggest that using a chlorella-derived dietary supplement attenuates the reduced salivary SIgA secretion during a training camp for a competitive sport like *kendo*.

We investigated the immune-enhancing effects of a chlorella-derived dietary supplement by determining the salivary SIgA secretion rates. Salivary SIgA is the first line of defense against respiratory tract infections, such as pneumonia and influenza [1]. Recently, Yamauchi *et al.*[4] investigated salivary SIgA secretion rates, Epstein–Barr virus (human herpes virus) DNA levels in saliva, and the number of upper respiratory symptoms during a 1-month training camp for rugby football. They found that the salivary SIgA secretion rate was reduced on the day before the first expression of Epstein–Barr virus DNA and that the number of symptoms increased after DNA expression. Fahlman *et al.*[25] also reported that the salivary SIgA secretion rates were lower and the percent of subjects with upper respiratory tract infections was higher among university football players after they completed 6 weeks of very intensive training or 10 weeks of intercollegiate competition as compared to the beginning of the season and a period of rest and to a non-varsity group. The salivary SIgA concentration is also a good index for mucosal immune function. Gleeson *et al.*[26] reported that the mean salivary SIgA concentration during a 7-month training period for elite swimmers was inversely related to the number of infections contracted. However, salivary SIgA concentrations are unsuitable for investigating any acute effects of exercise training because exercise can affect the saliva flow rate, as demonstrated in this study. Indeed, Gleeson *et al.*[26] reported that salivary SIgA concentrations before an exercise session were related to the numbers of infections, but the concentrations immediately after the session were not. In light of this, we used the salivary SIgA secretion rate as an index for immunological depression induced by the *kendo* training camp.

In a previous study, we found that the salivary SIgA secretion rate increased after 4-week intake of a chlorella-derived supplement and that this increase derived primarily from the salivary SIgA concentration [9]. However, in the present study, we found no differences in the salivary SIgA secretion rates and concentrations before the camp between the placebo and chlorella groups. Because the salivary SIgA secretion rates before the camp, even in the placebo group, were higher than that we observed in our previous study [9], the potential for improving the salivary SIgA secretion might have been minimal. In addition, the camp-related decease in salivary SIgA secretion and its attenuation due to chlorella intake were due to changes in saliva flow rates. A possible reason is that a reduced salivary SIgA concentration was offset by a decrease in the saliva flow rate, which could result in no change in salivary SIgA concentrations. *Bogu* (*i.e.*, protective armor) obstructs the skin surface for evaporation and leads to heat accumulation [27]. A greater transpiration of saliva during *kendo* practice than during rest may be a reason for the marked decrease in saliva flow rates. However, these are only speculations. Additional studies will be needed to account for the mechanisms responsible for these discrepancies.

Because saliva samples were collected from the end of the morning training session to lunch during the camp, we consider that the acute and cumulative effects were included in the reduced salivary SIgA secretion during the camp. It is possible that reductions in salivary SIgA secretion after high-intensity exercise are mediated by a cytokine secretory function of T-cells via their expression of polymeric Ig receptors (pIgR) and by autonomic nerve activity via saliva secretion as a vehicle for SIgA. Kimura *et al.*[28] showed that a brief round of intense exercise resulted in reducing the expression of pIgR mRNA in the submandibular gland, which paralleled a reduced salivary SIgA concentration. In the present study, the saliva flow rate reductions during the training camps were greater than the decreases in salivary SIgA concentrations. Thus, it is possible that the effects of chlorella intake on salivary SIgA secretion during the training camp may be more closely related to autonomic nerve regulation than to T-cell function.

Immune function is related to various nutrients, such as proteins [17-19], vitamins [20-22], iron [23], and folate [24]. With regard to proteins, the SIgA concentrations in saliva and nasal secretions were reportedly lower in protein-calorie malnourished children than in healthy children [17,19]. However, this improved with nutritional support in a hospital [18]. Vitamin A deficiency has also been shown to decrease SIgA concentrations in the saliva [20] and intestinal fluids [21] of experimental animals. In addition, a combined deficiency of pyridoxine, a vitamin B6 compound, and pantothenic acid reportedly resulted in decreased antibody titers in human sera [22]. From a multicomponent analysis of the chlorella tablets we used, all of these nutrients were detected. Other nutrients that showed possible immune-enhancing effects in previous studies were also detected in chlorella tablets. Unfortunately, in this study, we could not determine which nutrient(s) attenuated the reduced salivary SIgA secretion during the training camp. However, it is reasonable that a wide spectrum of nutrients contributed to maintaining salivary SIgA secretion during the camp, as the individual components were not taken in large doses.

Various dietary supplements have been tested for their ability to attenuate immunological depression following intensive exercise. However, the reported effects of most nutrients were either negative or controversial; only two nutrients, carbohydrate and quercetin, have been recommended by the international society that specializes in exercise immunology [6]. Moreover, use of carbohydrate supplements before and/or during prolonged exercise has little effect on salivary SIgA secretion [6]. The effect of quercetin on SIgA secretion remains unclear [29,30]. Thus, dietary supplements that can attenuate the reduced SIgA secretion during a training camp have yet to be proposed. In this study, the chlorella-derived multicomponent supplement attenuated the decrease in salivary SIgA secretion during the *kendo* training camp, which suggests that supplementation with a mixture of various nutrients may be an efficient method to lower the infection risk in athletes. However, there may be a better composition for infection control in athletes. The next step will be to investigate changes in the serum concentration of nutrients that can affect SIgA secretion and determine the mechanisms involved in the observed effect of chlorella intake in order to determine the best nutritional composition of a dietary supplement for athletes.

This study had several limitations. First, there were differences in the conditions between the spring and summer camps (*e.g.*, number of days and afternoon work-out times). Also, it is possible that the eating habits of our subjects changed during the experimental period and were different between the spring and summer trials. However, we randomly divided our subjects into (1) a spring camp with placebo and summer camp with chlorella group and (2) a spring camp with chlorella and summer camp with placebo group. In addition, we asked the subjects not to change their eating habits during the respective experimental periods (*i.e.*, from the beginning of tablet intake to post-camp saliva sampling). Thus, we consider that the effects of the differences in the conditions between the spring and summer camps on our inter-group comparisons were not remarkable. Second, we calculated saliva flow rates based on mass rather than measuring the actual volume. Because the reported specific gravity of saliva in healthy young humans is approximately 1.003, the saliva flow rate and the salivary SIgA secretion rate would have been only slightly overestimated [31,32]. However, the body masses of our subjects did not decrease during the camps compared with their baseline values. A previous study reported that an exercise-induced loss of body mass of 1.1% did not change saliva osmolality [33]. Thus, it was possible to compare these indices obtained before the camp with those during and after the camp. Finally, it is possible that the timing of saliva sampling (before breakfast or before lunch) affected salivary SIgA secretion. A previous study demonstrated that the salivary SIgA concentration was not stable and was higher during the 2 h after awaking, especially during the first 30 min, compared to 3 to 10 h later [34]. However, the tendencies for our samples collected only before and during the summer camp (*i.e.*, samples obtained with the same timing schedule) were nearly identical to those for all samples.

## Conclusion

We conclude that using a chlorella-derived multicomponent supplement attenuates the reduced salivary SIgA secretion during training camp for a competitive sport. This effect appears to be due mainly to attenuating the decrease in the saliva flow rate and not by changes in salivary SIgA concentrations.

## Competing interests

SunChlorella Co., Ltd. provided funding for the study and supplied the test supplements used in the study. TO has received speaker's honorarium from SunChlorella Co., Ltd. KS, MI and IK have no competing interests.

## Authors’ contributions

TO and KS designed the research. TO, KS and MI conducted the research. TO performed the statistical analysis and wrote this paper with support from KS, MI and IK. All authors read and approved the final manuscript.
